# 1, 25-dihydroxy-vitamin D3 with tumor necrosis factor-alpha protects against rheumatoid arthritis by promoting p53 acetylation-mediated apoptosis via Sirt1 in synoviocytes

**DOI:** 10.1038/cddis.2016.300

**Published:** 2016-10-20

**Authors:** Xin Gu, Bingjie Gu, Xianhui Lv, Zhenzhen Yu, Rong Wang, Xiaoli Zhou, Wanxin Qiao, Zhiyuan Mao, Guoping Zuo, Qing Li, Dengshun Miao, Jianliang Jin

**Affiliations:** 1 Research Centre for Bone and Stem Cells, Department of Human Anatomy, Histology and Embryology, Nanjing Medical University, Nanjing, Jiangsu 211166, China; 2 Department of Rheumatology and Immunology, Nanjing First Hospital; Nanjing Medical University, Nanjing, Jiangsu 210000, China; 3 Jiangsu Jiankang Vocational College, Nanjing, Jiangsu 210029, China

## Abstract

Impaired apoptosis of fibroblast-like synoviocytes (FLSs) causes synovial hyperplasia, facilitating destruction of cartilage and bone in rheumatoid arthritis (RA). Tumor necrosis factor (TNF)-*α*, a dominant inflammatory mediator in RA pathogenesis, promotes progression of RA symptoms. Prevalence of 1, 25-dihydroxy-vitamin D_3_ (hereafter termed VD) deficiency is 30–63% in patients with RA. Whether VD leads to apoptosis or enhances TNF-*α*-mediated apoptosis in FLSs to ameliorate RA is unclear. To determine this, 10-week-old *CYP27B1-*deficient (*CYP27B1*^−/−^) mice with collagen-induced arthritis (CIA) were intraperitoneally treated with 1 *μ*g/kg VD every other day for 9 weeks. RA phenotypes were compared between vehicle-treated *CYP27B1*^−/−^ and wild-type CIA mice. Human rheumatoid FLS-MH7A cells were treated with Dulbecco's modified Eagle's medium (DMEM) without fetal bovine serum (FBS) for 24 h, then with different concentrations of VD and TNF-*α*, human vitamin D receptor (VDR) siRNA or the p53 pro-apoptotic inhibitor pifithrin-*α*. Apoptosis and p53 pro-apoptotic signaling were analyzed. The 19-week-old vehicle-treated *CYP27B1*^−/−^ CIA mice had increased cumulative arthritis scores and levels of serous rheumatoid factors and C-reactive protein. They had exacerbated articular cartilage and bone destruction, joint space narrowing, joint stiffness, deformity and dysfunction, synovitis and TNF-*α* secretion, FLS hyperplasia with increased proliferation and decreased apoptosis compared to CIA mice. These RA phenotypes that were aggravated in CIA mice by *CYP27B1* deficiency were largely rescued by VD treatment. *In vitro*, VD with TNF-*α* treatment upregulated p53 acetylation-mediated apoptosis in MH7A cells by promoting Sirt1 translocation from the nucleus to the cytoplasm. These findings indicated that VD with TNF-*α* protected against RA by promoting apoptosis of FLSs. The results indicated that clinical administration of VD could be a specific therapy to promote FLS apoptosis and prevent RA progression.

Rheumatoid arthritis (RA) is chronic synovial inflammation and fibroblast-like synoviocytes (FLSs) hyperplasia with subsequent destruction of articular cartilage and bone, joint swelling and space narrowing, and joint stiffness, deformity and dysfunction. These are the main pathological features of autoimmune diseases, which mostly invade multiple small, symmetrical joints of the hands and feet. RA affects up to 1% of adults worldwide.^1, 2, 3^ FLSs, in particular, are key in RA because they produce cytokines that perpetuate inflammation and proteases.^4^ Impaired apoptosis of FLSs is mainly the result of abnormal p53 pro-apoptotic signaling that results in changes in the composition and structure of the inflamed synovial membrane.^5, 6^ These changes cause the development of synovial hyperplasia and extend the life span of these FLSs, facilitating the destruction of cartilage and bone in RA.^3, 4, 7^

A previous clinical investigation showed that tumor necrosis factor-alpha (TNF-*α*), a dominant inflammatory mediator in RA pathogenesis, is highly expressed in synovial fluid in RA. Selectively blocking TNF-*α* alleviates the progression of RA symptoms.^8, 9^ However, whether TNF-*α* mediates anti-apoptosis or pro-apoptosis pathogenic responses in RA-FLSs is unknown.^10, 11^ Previous evidence supports that TNF-*α* inhibits pro-apoptosis by Bcl-2 family members in RA-FLS.^7^ However, several lines of evidence suggest that the binding of TNF-*α* to its cell surface receptor TNF-R1 could induce pro-apoptotic responses to FLSs. Methods for enhancing the TNF-*α*-mediated apoptotic effects on RA FLSs have not been determined.^11, 12^

The compound VD is considered to maintain homeostasis of calcium–phosphorus metabolism.^13^ However, recent studies suggest that VD may be important in preventing autoimmune diseases.^13, 14, 15^ Emerging clinical epidemiology evidence indicates that the prevalence of VD deficiency is 30-63% in people with RA, serum VD levels negatively correlate with RA activity, and VD supplements are a beneficial RA treatment.^2, 14^ Data show that VD upregulates TNF-*α*-mediated pro-apoptotic effects in prostate tumor cells and osteoblasts and down-regulates pro-inflammatory effects in RA macrophages and T cells.^12, 16^ However, whether VD leads to apoptosis or enhances TNF-*α*-mediated apoptosis in FLSs to ameliorate RA is unclear.

The pathological features of collagen-induced arthritis (CIA) in mice are consistent with typical pathological alterations in RA patients. Therefore, CIA is the most widely studied RA model.^9^

We induced CIA in *CYP27B1-*deficient (*CYP27B1*^−/−^) mice, and intraperitoneally injected them with 1 *μ*g/kg VD every other day. Their RA phenotypes were compared with vehicle-treated *CYP27B1*^−/−^ and wild-type (WT) CIA mice. We treated human rheumatoid FLS-MH7A cells with different concentrations of VD, TNF-*α* and human VDR siRNA and the p53 pro-apoptotic inhibitor pifithrin-*α*. Apoptosis and p53 pro-apoptotic signaling were analyzed.

## Results

### Levels of serum VD in VD-treated *CYP27B1*^−/−^ CIA mice restored to normal

Radioimmunoassay showed that serum VD levels in 10-week-old *CYP27B1*^−/−^ mice or 19-week-old vehicle-treated *CYP27B1*^−/−^ CIA mice were significantly decreased compared to those in 10-week-old WT or 19-week-old WT CIA mice, respectively. We confirmed that serum VD levels of 19-week-old VD-treated *CYP27B1*^−/−^ CIA mice were restored to normal (Supplementary Figure S1A).

To compare the serum VD levels between VD-treated WT mice and vehicle-treated WT mice, 10-week-old male WT mice were intraperitoneally injected with 1 *μ*g/kg VD or vehicle every other day for 3 weeks. The levels of serum VD did not differ between 13-week-old VD-treated and vehicle-treated WT mice (Supplementary Figure S1A). It demonstrated that VD supplement did not increase serum level of VD in healthy individuals with sufficient VD.

### Clinical indexes of rheumatoid arthritis were ameliorated in *CYP27B1*^*−/−*^ CIA mice by VD

**Figure 1:**
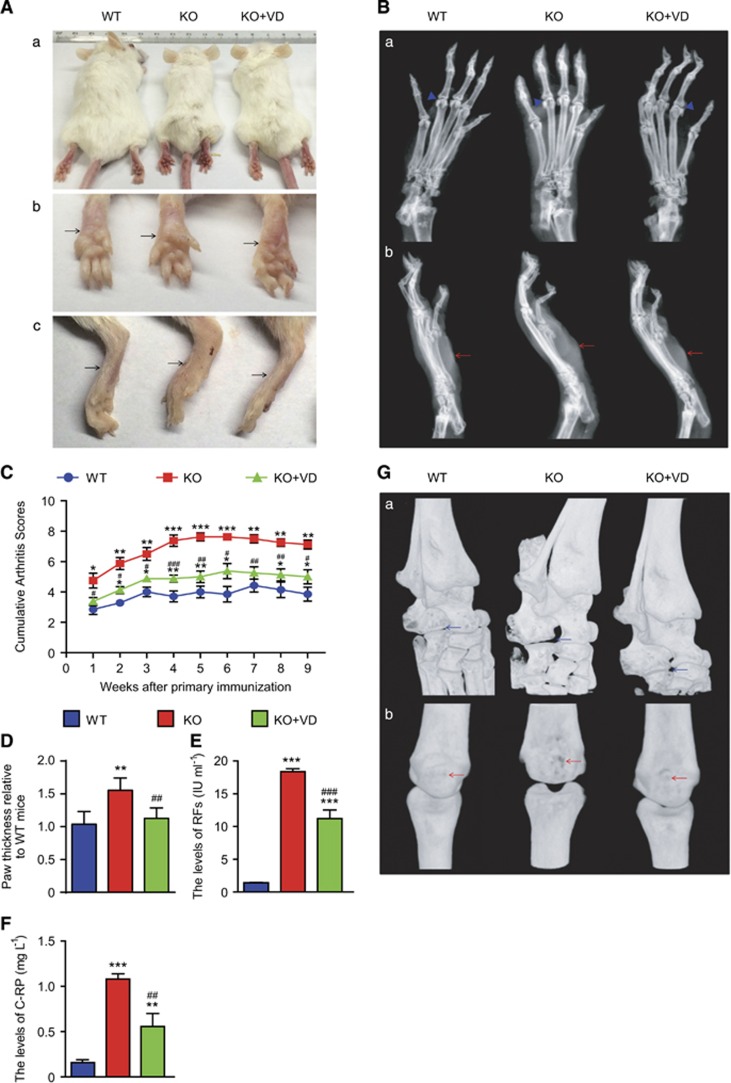
VD improved clinical indexes of rheumatoid arthritis in *CYP27B1*^−/−^ CIA mice. (**Aa**) Representative appearance and (**Ab**) underside and (**Ac**) side of arthritic hind palms from 19-week-old wild type (WT), *CYP27B1*^−/−^ (KO) and KO+VD CIA mice. Black arrows: hind palms. (**b**) Representative radiographs of (**Ba**) underside and (**Bb**) side of arthritic hind palms. Blue arrowhead: interphalangeal joints of arthritic hind palms; red arrow: hind palms. (**c**) Cumulative arthritis scores for subjective evaluation of arthritis severity of arthritic hind palms from 1 to 9 weeks after primary immunization. (**d**) Paw thickness relative to WT mice. (**e**) Levels of rheumatoid factors (RFs) and (**f**) C-reactive protein (C-RP) in serum determined by spectrophotometry. Values are mean±S.E.M. of six determinations per group. *P<0.05; **P<0.01; ***P<0.001 compared with WT group at the same age. ^#^*P*<0.05; ^##^*P*<0.01; ^###^*P*<0.001 compared with KO group at the same age. (**g**) Representative 3D-reconstructed images of (**Ga**) intertarsal joint and (**Gb**) interphalangeal joints of arthritic hind palms. Blue arrow: deformity of intertarsal joints; red arrow: bone erosion of phalanxes

To assess if VD treatment ameliorated clinical indexes of RA in *CYP27B1*^−/−^ CIA mice, arthritis severity, swelling degree, rheumatoid serum biochemical measurements, and bone and joint erosion of arthritic hind palms were subjectively evaluated in 19-week-old, VD-treated *CYP27B1*^−/−^ CIA, vehicle-treated *CYP27B1*^−/−^ and WT CIA mice. Compared with WT CIA mice, body size was decreased; however, cumulative arthritis scores from 1 to 9 weeks after primary immunization, paw thickness, levels of rheumatoid factors (RFs) and C-reactive protein (C-RP) from serum increased significantly in vehicle-treated *CYP27B1*^−/−^ CIA mice (Figures 1a–f). X-ray and three-dimensional (3D) reconstructed graphs of hind palms showed that metatarsophalangeal joints were characterized by blurred articular surface, cartilage erosion and narrowed articular space on radiographs; intertarsal joints showed deformity and phalanxes were eroded on 3D-reconstructed graphs (Figures 1b and g). Compared with vehicle-treated *CYP27B1*^−/−^ CIA mice, body size was increased; however, cumulative arthritis scores from 1 to 9 weeks after primary immunization, paw thickness, and RFs and C-RP serum levels decreased significantly in VD-treated *CYP27B1*^−/−^ mice (Figures 1a–f). The VD treatment ameliorated the arthritic characteristics of metatarsophalangeal joints and intertarsal joints, and decreased bony erosion of phalanxes in VD-treated *CYP27B1*^−/−^ CIA mice (Figures 1b and g).

### Histological bone destruction, cartilage erosion and synovial inflammation of rheumatoid arthritis were ameliorated in *CYP27B1*^−/−^ CIA mice by VD

**Figure 2:**
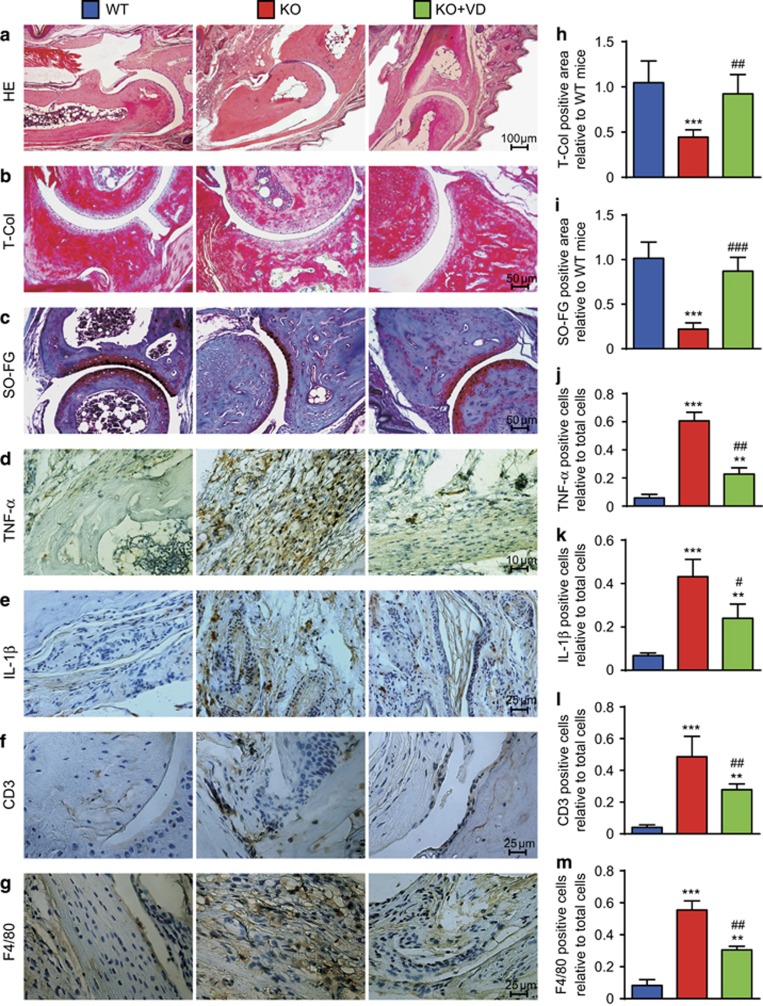
VD improved histological bone destruction, cartilage erosion and synovial inflammation of rheumatoid arthritis in *CYP27B1*^−/−^ CIA mice. (**a**) Representative micrographs of interphalangeal joints sections stained with HE from 19-week-old wild type (WT), *CYP27B1*^−/−^ (KO) and KO+VD CIA mice. Representative micrographs of paraffin-embedded sections stained histochemically for (**b**) total collagen (T-col) and (**c**) Safranin O and fast green double dyeing (SO-FG). Representative micrographs of paraffin-embedded sections stained immunohistochemically for (**d**) TNF-*α*, (**e**) IL-1*β*, (**f**) CD3 and (**g**) F4/80. (**h**-**i**) T-Col-positive and SO-FG-positive areas relative to WT mice in sections stained for figures **b**–**c**. Percentage of cells positive for (**j**) TNF-*α*, (**k**) IL-1*β*, (**l**) CD3 or (**m**) F4/80 relative to total cells in sections stained for figures **d**–**g**. Values are mean±S.E.M. of six determinations of each group. **P<0.01; ***P<0.001 compared with WT. ^#^*P*<0.05; ^##^*P*<0.01; ^###^*P*<0.001 compared with KO

To investigate if VD treatment ameliorated histological bone destruction, cartilage erosion and synovial inflammation of RA in *CYP27B1*^−/−^ CIA mice, HE, total collagen, and Safranin O and fast green double dyeing (SO-FG) histochemical staining were performed to analyze trabecular and cartilage volume. TNF-*α*, IL-1*β*, CD3 and F4/80 immunohistochemical staining for synovial inflammation were performed. Compared with WT CIA mice, trabecular and cartilage bone volume decreased significantly; however, percentages of TNF-*α*, IL-1*β*, CD3 and F4/80 positive inflammatory cells in FLSs increased in vehicle-treated *CYP27B1*^−/−^ CIA mice (Figure 2). Compared with vehicle-treated *CYP27B1*^−/−^ CIA mice, trabecular and cartilage bone volume increased significantly; however, percentages of TNF-*α*, IL-1*β*, CD3 and F4/80-positive inflammatory cells in synovial membranes decreased in VD-treated *CYP27B1*^−/−^ CIA mice (Figure 2).

### Synovial hyperplasia caused by increased FLS proliferation and decreased apoptosis were ameliorated in *CYP27B1*^−/−^ CIA mice by VD

**Figure 3:**
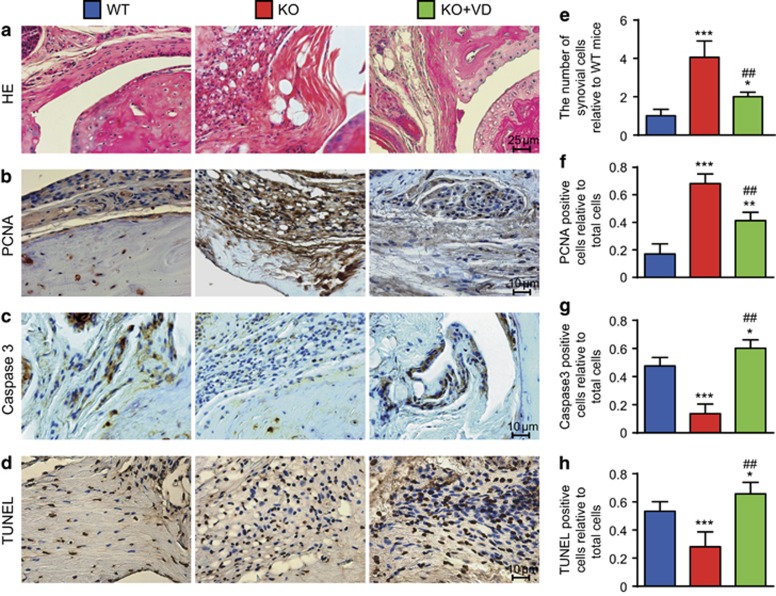
VD decreased proliferation and increased apoptosis of FLSs in *CYP27B1*^−/−^ CIA mice. Representative micrographs of sections of paraffin-embedded interphalangeal joints from 19-week-old wild type (WT), *CYP27B1*^−/−^ (KO) and KO+VD CIA mice stained for (**a**) HE, and immunohistochemically for (**b**) PCNA, (**c**) Caspase3 and (**d**) TUNEL. (**e**) The number of FLSs relative to WT mice was determined in HE-stained sections. The percentage of (**f**) PCNA-positive, (**g**) Caspase3-positive and (**h**) TUNEL-positive cells relative to total cells was determined in stained sections in **b**–**d**. Values are mean±S.E.M. of six determinations per group. *P<0.05; **P<0.01; ***P<0.001 compared with WT. ^##^*P*<0.01 compared with KO

To determine if VD treatment ameliorated synovial hyperplasia in *CYP27B1*^−/−^ CIA mice, HE staining was used for comparisons with WT CIA mice. The number of synovial cells increased in vehicle-treated *CYP27B1*^−/−^ mice. Compared with vehicle-treated *CYP27B1*^−/−^ CIA mice, the number of synovial cells decreased in VD-treated *CYP27B1*^−/−^ CIA mice (Figures 3a and e).

To further determine if VD treatment decreased proliferation and increased apoptosis of FLSs in *CYP27B1*^−/−^ CIA mice, proliferating cell nuclear antigen (PCNA) immunohistochemistry staining was used to detect proliferation, and Caspase3 staining and TUNEL assays were used to analyze apoptosis. Compared to WT CIA mice, the percentage of PCNA-positive FLSs decreased and percentages of Caspase3-positive and TUNEL-positive FLSs significantly increased in *CYP27B1*^−/−^ CIA mice; the percentage of PCNA-positive FLSs increased with a significant increase in percentages of Caspase3-positive and TUNEL-positive FLSs in VD-treated *CYP27B1*^−/−^ CIA mice. Compared with vehicle-treated *CYP27B1*^−/−^ CIA mice, the percentage of PCNA-positive FLSs decreased and percentages of Caspase3-positive and TUNEL-positive FLSs significantly increased in VD-treated *CYP27B1*^−/−^ CIA mice (Figures 3b–d and f–h).

### Apoptosis of human rheumatoid FLSs increased by VD with TNF-*α*

**Figure 4:**
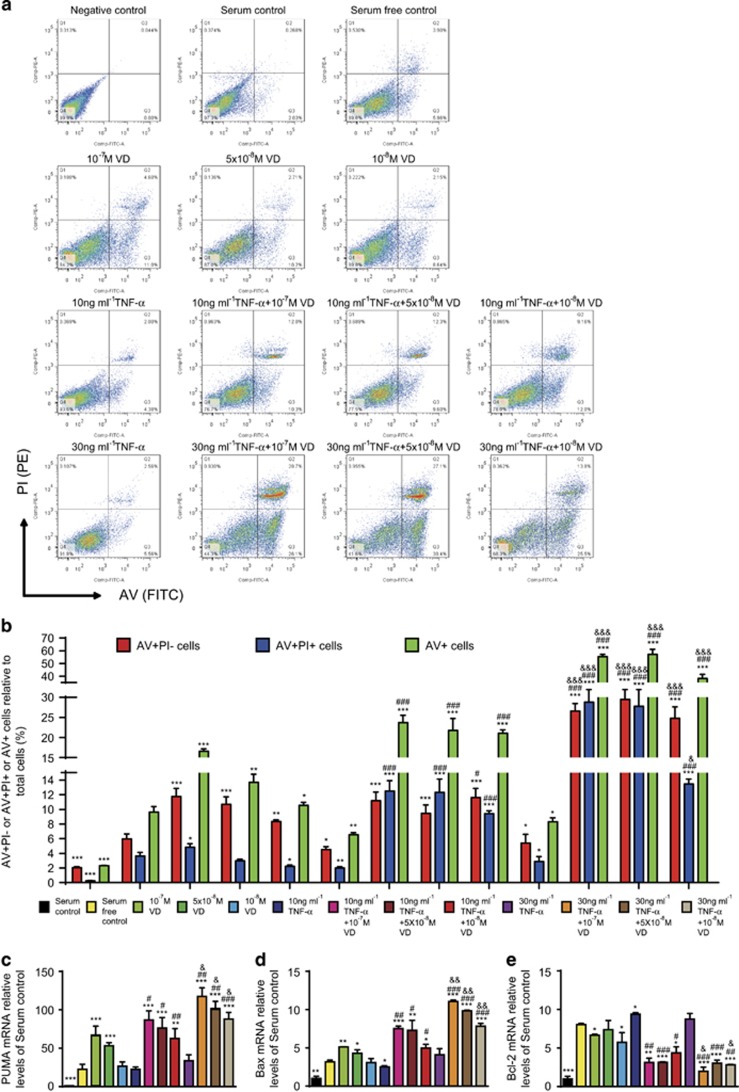
VD with TNF-*α* promoted apoptosis of rheumatoid FLSs. Human rheumatoid FLS-MH7A cells were treated with DMEM and 10% FBS (serum control), DMEM (serum-free control), DMEM and indicated concentrations of VD with or without TNF-*α*. (**a**) Flow cytometry of double-stained cells using annexin V (AV) and propidium iodide (PI). (**b**) AV-positive but PI-negative cells (AV+PI–), AV and PI double-positive cells (AV+PI+), and total AV-positive cells (AV+) were quantified. Values are mean±S.E.M. of six determinations per group. *P<0.05; **P<0.01; ***P<0.001 compared to serum-free control. ^#^*P*<0.05; ^###^*P*<0.001 compared with the same concentration of VD. ^&^*P*<0.05; ^&&&^*P*<0.001 compared with 10 ng/ml TNF-*α* and the same concentration of VD. (**c**) *PUMA*, *Bax* and *Bcl-2* mRNA by group by real-time RT-PCR, calculated as ratio to *GAPDH* mRNA, expressed relative to serum control. *P<0.05; **P<0.01; ***P<0.001 compared with serum-free control. ^#^*P*<0.05; ^###^*P*<0.001 compared with the same concentration of VD. ^&^*P*<0.05; ^&&&^*P*<0.001 compared with 10 ng/ml TNF-*α* and the same concentration of VD

To investigate if VD with TNF-*α* promoted apoptosis of rheumatoid FLSs, human rheumatoid FLS-MH7A cells were treated with different concentrations of VD and/or TNF-*α*. Apoptosis was detected by AV and PI double staining and flow cytometry. Results are shown in detail in Table 1. These results demonstrated that with TNF-*α* treatment at the corresponding concentration, VD supplementation significantly increased the apoptosis of rheumatoid FLSs. Moreover, the pro-apoptotic effect of VD was increased with elevated concentrations of TNF-*α* (Figures 4a and b).

To detect further expression of pro-apoptotic and anti-apoptotic genes, real-time RT-PCR were performed for Bcl-2 binding component 3 (also known as p53 upregulated modulator of apoptosis; *PUMA*), Bcl-2-associated X (*Bax*) protein and *Bcl-2* (Table 1). These results demonstrated that with TNF-*α* treatment at the corresponding concentration, VD supplementation significantly increased expression of pro-apoptotic genes and decreased expression of anti-apoptotic genes in rheumatoid FLSs. Moreover, under VD treatment at the corresponding concentration, expression of pro-apoptotic genes was increased with TNF-*α* concentration. Expression of anti-apoptotic genes was decreased with increased TNF-*α* concentration (Figures 4c–e).

### Human rheumatoid FLS apoptosis after VD with TNF-*α* was mediated by VDR and p53 pro-apoptotic signaling

To further investigate if apoptosis of rheumatoid FLSs induced by VD with TNF-*α* treatment was mediated by VDR and p53 pro-apoptotic signaling, human rheumatoid FLS-MH7A cells were knocked down with VDR siRNA.

Compared to negative control (NC) siRNA, VDR gene expression was downregulated to 17.87% in cells with VDR siRNA1, 52.52% in cells with VDR siRNA2 and 30.24% in cells with siRNA3 (Supplementary Figure S1C). *β*-actin gene expression was downregulated to 36.4% in cells with *β*-actin siRNA, but was not altered in cells transfected with VDR siRNA1, 2 or 3 (Supplementary Figure S1D). VDR siRNA1 was for subsequent experiments. VDR protein expression was downregulated to 34.19% in cells with VDR siRNA1 compared to NC siRNA (Supplementary Figures S1E and S1F).

**Figure 5:**
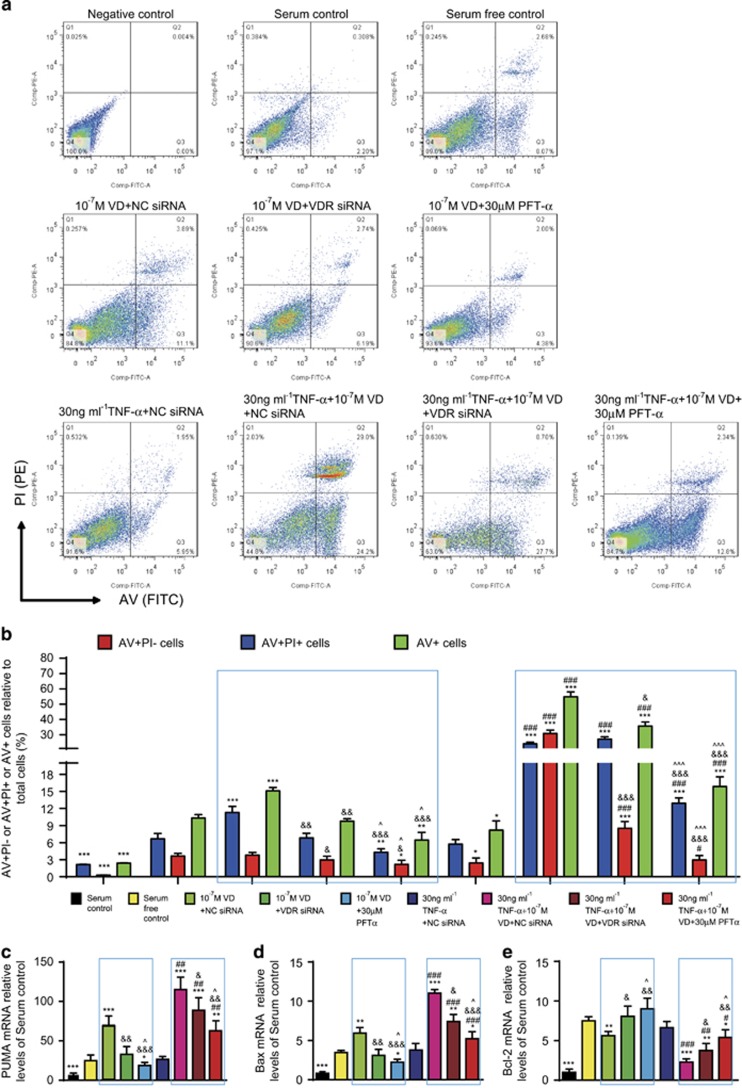
VD with TNF-*α* induced apoptosis of rheumatoid FLSs through VDR and p53 pro-apoptotic signaling. Human rheumatoid FLS-MH7A cells were treated with DMEM and 10% FBS (serum control), DMEM (serum-free control), DMEM and 10^–7^ M VD and VDR-negative control small interfering RNA (10^–7^ M VD+NC siRNA), DMEM and 10^–7^ M VD and VDR siRNA (10^–7^ M VD+VDR siRNA), DMEM and 10^–7^ M VD and 30 *μ*M pifithrin-*α* (PFT-*α*) (10^–7^ M VD+30 *μ*M PFT-*α*), DMEM and 30 ng/ml TNF-*α* and NC siRNA (30 ng/ml TNF-*α* + NC siRNA), DMEM and 30 ng/ml TNF-*α* and 10^–7^ M VD and NC siRNA (30 ng/ml TNF-*α* + 10^–7^ M VD+NC siRNA), DMEM and 30 ng/ml TNF-*α* and 10^–7^ M VD and 30 *μ*M PFT-*α* (30 ng/ml TNF-*α* + 10^–7^ M VD+30 *μ*M PFT-*α*). (**a**) Flow cytometry of cells double-stained for annexin V (AV) and propidium iodide (PI). (**b**) AV-positive but PI-negative cells (AV+PI–), AV and PI double-positive cells (AV+PI+) and total AV-positive cells (AV+) were quantified. Values are mean±S.E.M. of six determinations per group. *P<0.05; **P<0.01; ***P<0.001 compared with serum-free control. ^#^*P*<0.05; ^###^*P*<0.001 compared with 10^–7^ M VD but not the TNF-*α*-treated group. ^&^*P*<0.05; ^&&^*P*<0.01; ^&&&^*P*<0.001 compared with 10^–7^ M VD and NC siRNA with or without 30 ng/ml TNF-*α* inside the same blue box. ^*P*<0.05; ^^^*P*<0.001 compared with 10^–7^ M VD and VDR siRNA with or without 30 ng/ml TNF-*α* inside the same blue box. (**c**) Relative levels of *Puma*, *Bax* and *Bcl-2* mRNA by group by real-time RT-PCR, calculated as ratio to *GAPDH* mRNA, expressed relative to serum control. Values are mean±S.E.M. of six determinations per group. *P<0.05; **P<0.01; ***P<0.001 compared with serum-free control. ^#^*P*<0.05; ^##^*P*<0.01; ^###^*P*<0.001 compared with 10^–7^ M VD but not the TNF-*α*-treated group. ^&^*P*<0.05; ^&&^*P*<0.01; ^&&&^*P*<0.001 compared with 10^–7^ M VD and NC siRNA with or without 30 ng/ml TNF-*α* inside the same blue box. ^*P*<0.05 compared with 10^–7^ M VD and VDR siRNA with or without 30 ng/ml TNF-*α* inside the same blue box

Cells were treated with VD and/or TNF-*α* and p53 pro-apoptotic inhibitor PFT-*α*, and apoptosis detected by AV and PI double staining by flow cytometry. Detailed results are shown in Table 2. The pro-apoptotic effect induced by VD was attenuated when VDR was knocked down or p53 was inhibited. Moreover, attenuation of the pro-apoptotic effect was more obvious in the p53-inhibited group than in VDR-knocked down group (Figures 5a and b).

To detect further expression of pro-apoptotic and anti-apoptotic genes, real-time RT-PCR was performed for *PUMA*, *Bax* and *Bcl-2* (Table 2). When VDR was knocked down or p53 was inhibited, expression of the upregulated pro-apoptotic genes induced by VD was decreased, and expression of the downregulated anti-apoptotic gene induced by VD was increased. Moreover, the above changes were more obvious in the p53-inhibited group than in VDR-knocked down group (Figures 5c–e).

### P53 acetylation-mediated apoptosis in human rheumatoid FLSs promoted by VD with TNF-*α*

**Figure 6:**
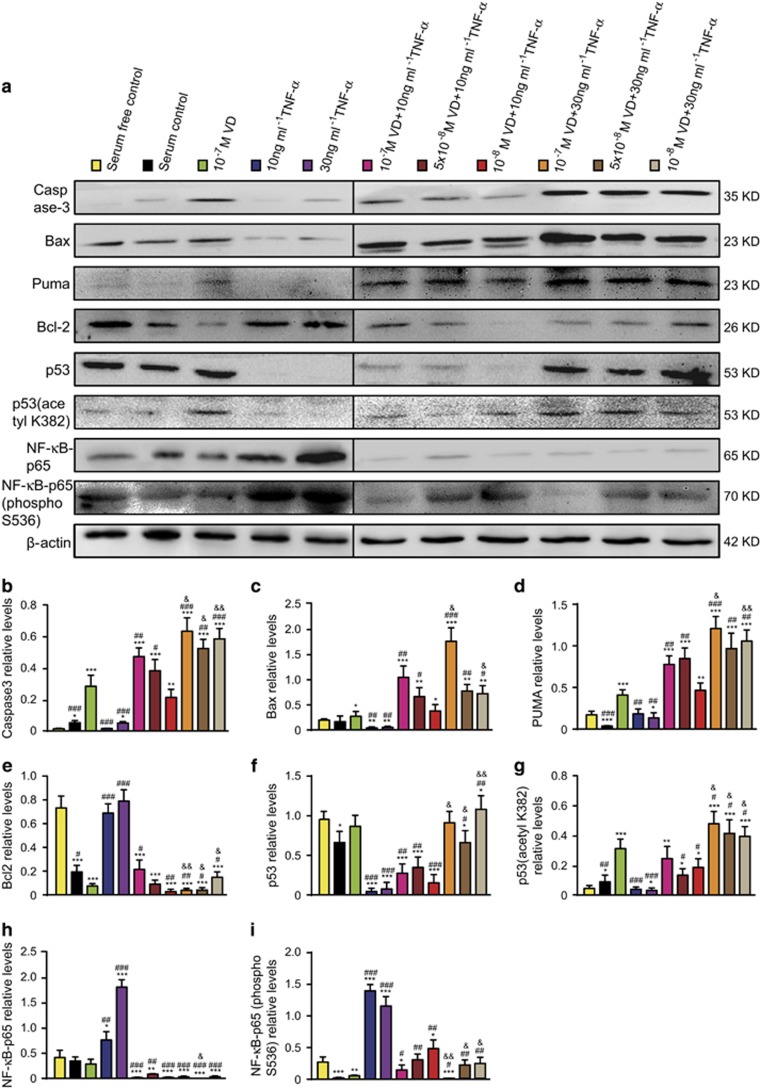
VD with TNF-*α* promoted p53 acetylation-mediated apoptosis in human rheumatoid FLSs. Human rheumatoid FLS-MH7A cells were treated with DMEM and 10% FBS (serum control), DMEM (serum-free control), DMEM and indicated concentrations of VD with or without TNF-*α*. (**a**) Western blots of cells by group for Caspase-3, Bax, Puma, Bcl-2, p53, p53 (acetyl K382), NF-*κ*B-p65 and NF-*κ*B-p65 (phospho Ser536). *β*-actin was the loading control. (**b**-**h**) Protein relative to *β*-actin was assessed by densitometry. Values are mean±S.E.M. of six determinations per group. *P<0.05; **P<0.01; ***P<0.001 compared with serum-free control. ^#^*P*<0.05; ^##^*P*<0.01, ^###^*P*<0.001 compared with 10^–7^ M VD. ^&^*P*<0.05; ^&&^*P*<0.01 compared with 10 ng/ml TNF-*α* and the same concentration of VD

To determine if VD with TNF-*α* promoted p53 acetylation-mediated apoptosis in rheumatoid FLSs, western blots were performed for p53 acetylation-mediated apoptosis-related molecules (Table 3). These results demonstrated that with TNF-*α* treatment at the corresponding concentration, VD supplementation significantly increased p53-acetylation-mediated apoptosis, including increased protein levels of Caspase 3, Bax, PUMA, p53 and p53 (acetyl K382), and decreased protein levels of Bcl-2. Moreover, with VD treatment at the corresponding concentration, p53-acetylation-mediated apoptosis increased with TNF-*α* concentration (Figures 6a–g).

To determine if VD with TNF-*α* inhibited p53-mediated pro-apoptotic effects by NF-*κ*B-p65 phosphorylation at Ser-536 in rheumatoid FLSs, western blotting was performed for expression of NF-*κ*B-p65 and NF-*κ*B-p65 (phospho S536) (Table 3). These results demonstrated that with TNF-*α* treatment at the corresponding concentration, VD supplementation significantly decreased expression of NF-*κ*B-p65 and NF-*κ*B-p65 (phospho S536). Moreover, with VD treatment at the corresponding concentration, expression of NF-*κ*B-p65 and NF-*κ*B-p65 (phospho S536) was decreased as TNF-*α* concentration increased (Figures 6a, h–i).

### Sirt1 nuclei to cytoplasm translocation is promoted in human rheumatoid FLSs by VD with TNF-*α*

To determine if VD with TNF-*α* treatment promoted Sirt1 translocation from nuclei to cytoplasm in rheumatoid FLSs, Sirt1 immunofluorescence was performed with sections of paraffin-embedded interphalangeal joints from CIA mice or MH7A cells. Sirt1 expression in nuclear or cytoplasm fractions of MH7A cells was detected by western blots.

**Figure 7:**
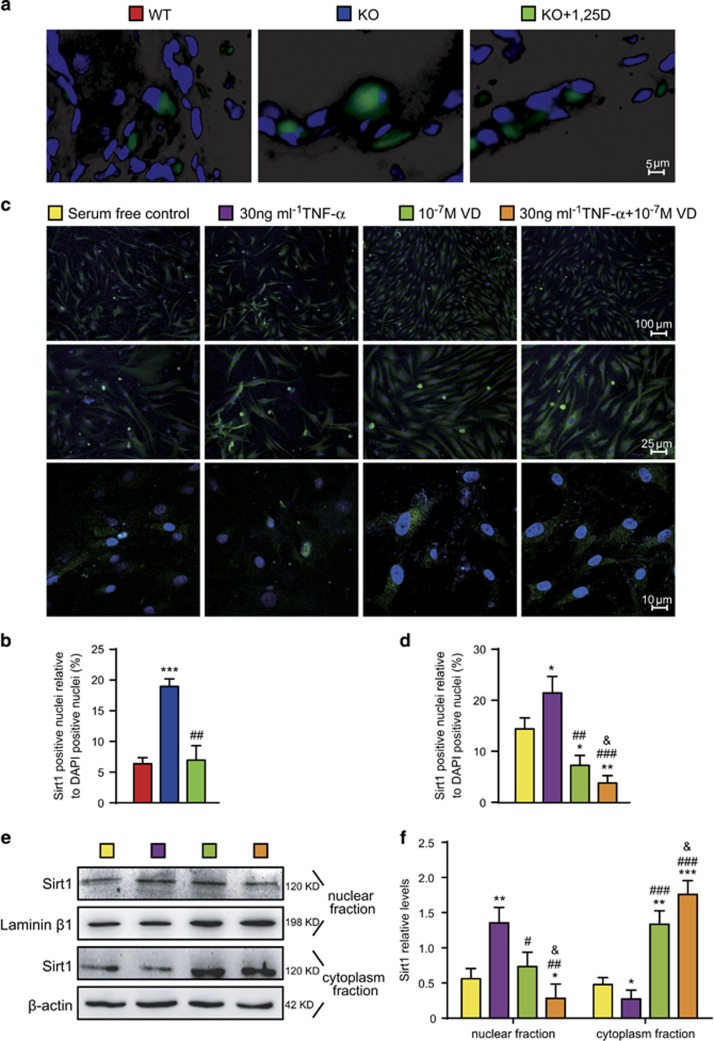
VD with TNF-*α* promoted SIRT1 translocation from nuclei to cytoplasm in human rheumatoid FLSs. (**a**) Representative micrographs of sections from paraffin-embedded interphalangeal joints from 19-week-old wild type (WT), *CYP27B1*^−/−^ (KO) and KO+VD mice immunofluoresence in FLSs for Sirt1 (green), DAPI for nuclei (blue). Values are mean±S.E.M. of six determinations per group. ***P<0.001 compared with WT. ^##^*P*<0.01 compared with KO. (**b**) Percentage of Sirt1-positive nuclei relative to DAPI-positive nuclei. (**c**) Human rheumatoid FLS-MH7A cells treated with DMEM (serum-free control), DMEM and 30 ng/ml TNF-*α* (30 ng/ml TNF-*α*), DMEM and 10^–7^ M VD (10^–7^ M VD), DMEM and 30 ng/ml TNF-*α* and 10^–7^ M VD (30 ng/ml TNF-*α* + 10^–7^ M VD). Representative micrographs of cells with immunofluoresence for Sirt1 (green) and DAPI for nuclei (blue). (**d**) Percentage of Sirt1-positive relative to DAPI-positive nuclei. (**e**) Western blots of MH7A cell nuclear fraction or cytoplasm fractions for Sirt1. Loading controls were Laminin *β*1 for the nuclear fraction and *β*-actin for the cytoplasm fraction. Values are mean±S.E.M. of six determinations per group. *P<0.05; **P<0.01; ***P<0.001 compared with serum-free control. ^#^*P*<0.05; ^##^*P*<0.01; ^###^*P*<0.001 compared with 30 ng/ml TNF-*α*. ^&^*P*<0.05 compared with 10^–7^ M VD

Compared with WT CIA mice, the percentage of Sirt1-positive nuclei relative to 4', 6-diamidino-2-phenylindole (DAPI) nuclei of synovial cells increased in vehicle-treated *CYP27B1*^−/−^ CIA mice. Compared with vehicle-treated *CYP27B1*^−/−^ CIA mice, the percentage of Sirt1-positive nuclei relative to DAPI nuclei of synovial cells decreased in VD-treated *CYP27B1*^−/−^ CIA mice (Figures 7a and b).

Compared with the serum-free control group, the percentage of Sirt1-positive nuclei relative to DAPI nuclei and Sirt1 protein in the nuclear fraction of MH7A cells increased in the 30 ng/ml TNF-*α* treatment group; both parameters significantly decreased with 10^–7^ M VD, or 10^–7^ M VD with 30 ng/ml TNF-*α* treatment. Compared with the 30 ng/ml TNF-*α* treatment, the percentage of Sirt1 positive nuclei relative to DAPI nuclei and Sirt1 protein in the nuclear fraction of MH7A cells significantly decreased with 10^–7^ M VD, or 10^–7^ M VD with 30 ng/ml TNF-*α* treatments. Compared with the 10^–7^ M VD-treatment group, the percentage of Sirt1-positive nuclei relative to DAPI nuclei and Sirt1 protein in the nuclear fraction of MH7A cells decreased with 10^–7^ M VD with 30 ng/ml TNF-*α* treatment (Figures 7c–f).

Compared with the serum-free control group, Sirt1 protein in the cytoplasmic fraction of MH7A cells decreased in the 30 ng/ml TNF-*α*-treatment group; it significantly increased with 10^–7^ M VD, or 10^–7^ M VD with 30 ng/ml TNF-*α* treatment. Compared with the group treated with 30 ng/ml TNF-*α*, Sirt1 protein in the cytoplasmic fraction of MH7A cells significantly increased with groups treated with 10^–7^ M VD, and 10^–7^ M VD with 30 ng/ml TNF-*α*. Compared with the 10^–7^ M VD treatment group, Sirt1 protein in the cytoplasmic fraction of MH7A cells increased in the group of 10^–7^ M VD and 30 ng/ml TNF-*α* treatments (Figures 7c–f).

These results demonstrated that VD with TNF-*α* promoted Sirt1 translocation from the nucleus to the cytoplasm in rheumatoid FLSs more than VD alone, and reversed the inhibitory effect of TNF-*α* on Sirt1 translocation from the nucleus to the cytoplasm.

### Apoptosis of normal FLSs were not induced by VD with TNF-*α*

To determine whether apoptosis of normal FLSs was induced by VD with TNF-*α*, normal FLSs were treated with the same treatment as MH7A cells. The percentages of AV-positive and PI-negative (AV+PI−), AV and PI double-positive (AV+PI+) and total AV-positive (AV+) normal FLSs in the serum-free control group were 34.87±1.62%, 6.60±1.48% and 41.47±1.69%, respectively (Supplementary Figures S2A and S2B). However, the percentages of AV+PI−, AV+PI+ and total AV+FLSs in the serum-free control group of MH7A cells were 5.99±0.65%, 3.66±0.47% and 9.65±0.73% respectively (Figures 4a and b). These results demonstrated that the resistance of normal FLSs to serum-free culture is poorer than MH7A cells, and easier to be induced into apoptosis. Previous studies support that normal FLSs appear less resistant to apoptosis than RA-FLSs.^4^ Thus, normal FLSs were treated with DMEM or DMEM and 10^–7^ M VD and/or 10 or 30 ng/ml TNF-*α* for 24 h. The percentages of AV+PI−, AV+PI+ and total AV+FLSs in the serum-free control group of normal FLSs were 5.0±0.75%, 6.78±0.71% and 11.78±0.25%, respectively (Supplementary Figures S2C and S2D). Compared with the serum-free control group, the percentages of AV+PI−, AV+PI+ or total AV+FLSs in the 10^–7^ M VD group, 10 ng/ml TNF-*α* group, 10 ng/ml TNF-*α* with 10^–7^ M VD group, 30 ng/ml TNF-*α* group, and 30 ng/ml TNF-*α* with 10^–7^ M VD group were no different (Supplementary Figures S2C and S2D).

## Discussion

In this study, we demonstrated that *CYP27B1* deficiency increased cumulative arthritis scores and levels of serous RFs and C-RP. *CYP27B1* deficiency exacerbated articular cartilage and bone destruction, joint space narrowing, joint stiffness, deformity and dysfunction, synovitis and TNF-*α* secretion. *CYP27B1* deficiency caused FLSs hyperplasia with increased proliferation and decreased apoptosis in CIA mice. These typical RA phenotypes in *CYP27B1* deficiency were largely rescued by VD treatment. *In vitro*, results demonstrated that VD with TNF-*α* treatment upregulated p53 acetylation-mediated apoptosis in human rheumatoid FLS-MH7A cells by promoting Sirt1 translocation from nuclei to cytoplasm. These findings indicated that VD with TNF-*α* protected against RA by promoting p53 acetylation-mediated apoptosis via Sirt1 translocation from nuclei to cytoplasm in RA-FLSs.

Previous studies suggest that FLS hyperplasia is a key to both initiation and the perpetuation of RA. FLS hyperplasia has been linked most prominently to the progressive destruction of articular structures, particularly cartilage.^7, 17^ The ability of hyperplastic FLSs to erode articular cartilage is a multistep process that includes attachment to cartilage and synthesis of proteolytic enzymes that degrade the extracellular matrix.^4^ A broad array of inflammatory mediators and cytokines are secreted by hyperplastic FLSs to induce apoptosis of articular cartilage.^4^ Hyperplastic FLS results in joint stiffness, deformity and dysfunction.^4^ Therefore, inhibiting inflammation and proliferation and promoting apoptosis of FLS is critical for RA treatment.

Previous observations demonstrate that TNF-*α* induces pro-inflammatory responses and upregulates TNF-*α* and IL-1*β* through activating the NF-*κ*B signaling pathway that stimulates FLS overgrowth to form a mass of synovial tissue.^11, 16, 18^ Our results revealed that *CYP27B1* deficiency upregulated expression of TNF-*α* and IL-1*β*, increased CD3-positive and F4/80-positive inflammatory cells and PCNA-positive FLSs. We found that VD downregulated expression of TNF-*α* and IL-1*β*, and decreased CD3-positive and F4/80-positive inflammatory cells and PCNA-positive FLSs in *CYP27B1*-deficient CIA mice. Results from a previous study support that NF-*κ*B-mediated pro-inflammatory gene transcription is repressed by VD, resulting in suppressed production of inflammatory cytokines such as IL-1*β* and TNF-*α*.^13^ Our study further demonstrated that VD and TNF-*α* treatment significantly inhibited NF-*κ*B-p65 expression in human rheumatoid FLSs. Thus, VD could be used for RA treatment as an anti-inflammatory and anti-proliferative molecule for hyperplastic FLSs.

Several lines of evidence suggest that impaired and inhibited apoptosis contributes to the accumulation of RA-FLSs, and is pivotal to RA-FLS-mediated disease progression.^17^ Therefore, finding pro-apoptotic molecules of hyperplastic FLSs is important for RA treatment. Our results revealed that *CYP27B1* deficiency decreased Caspase3-positive and TUNEL-positive FLSs. We also found that VD increased the percentages of Caspase3-positive and TUNEL-positive FLSs in *CYP27B1*-deficient CIA mice compared to WT CIA mice. The percentage of TNF-*α*-positive cells in synovial membranes increased in VD-treated *CYP27B1*-deficient CIA mice compared to WT CIA mice. Consistent with these results, VD might cooperate with TNF-*α* to promote apoptosis of RA-FLSs *in vivo*. Consistently, VD with TNF-*α* treatment significantly increased AV+PI–, AV+PI+ and AV+cells and Caspase3 protein in human rheumatoid FLSs *in vitro*. Thus, VD could also be a pro-apoptotic molecule in hyperplastic FLSs, in cooperation with TNF-*α*, to promote apoptosis for RA treatment.

As a critical tumor suppressor gene and apoptotic regulator implicated in RA, p53 induces cell apoptosis through posttranslational modification to upregulate downstream pro-apoptotic genes *Bax*, *PUMA* and *Noxa*. P53 also combines with the anti-apoptotic gene *Bcl-2* to inhibit anti-apoptotic effects.^4, 19^ Loss or inactivation of p53 caused by functional mutations has been universally demonstrated in RA-FLSs, most extensively in patients with advanced, destructive disease.^4, 20, 21^ P53 mutations increase apoptosis resistance and the level of inflammatory secretion in RA-FLSs.^4, 21^ We found that VD with TNF-*α* treatment increased Bax and PUMA and decreased Bcl-2 mRNA and protein in human rheumatoid FLSs. Moreover, PFT-*α*, a p53 pro-apoptotic inhibitor, decreased apoptosis of human rheumatoid FLSs induced by VD and TNF-*α*. Our results demonstrated that VD with TNF-*α* promoted apoptosis of human rheumatoid FLSs through upregulating p53 pro-apoptotic signaling and ameliorated the development of RA. Recent evidence suggests that PUMA induces apoptosis not only through a p53-dependent mechanism, but also through p53-independent signaling.^4, 22^ Consistently, our results suggested that PUMA was still expressed, even if PFT-*α* was supplied to inhibit p53 apoptotic signaling in human rheumatoid FLSs. Therefore, apoptosis of RA-FLSs induced by VD and TNF-*α* supplement might be partly mediated by p53-independent pro-apoptotic signaling; this hypothesis remains to be investigated.

P53 acetylation is a post-translational modification that enhances the ability of p53 to regulate downstream genes.^23^ Equilibrium of p53 acetylation is partly maintained by Sirt1. Sirt1 preferentially de-acetylates p53 at K382 to have a profound negative impact on the capacity of p53 to induce expression of target genes involved in apoptosis such as *PUMA* and *Bax*.^19^ The p53 de-acetylation caused by Sirt1 inhibits p53-dependent pro-apoptosis by increasing ubiquitination-mediated degradation of p53.^4, 24^ Evidence suggests that Sirt1 is highly expressed in RA-FLSs, which is positively related to RA severity.^24, 25^ Our study found that VD with TNF-*α* inhibited p53 de-acetylation, increased p53 acetylation and promoted p53-dependent pro-apoptosis.

NF-*κ*B is constitutively activated in RA.^26^ The anti-apoptogenic function of NF-*κ*B is well recognized, and is achieved through the gene expression of several anti-apoptogenic molecules, including *Bcl-xL*, *XIAP*, *cIAP1*, *cIAP2* and *TRAFs* induced by NF-*κ*B.^6^ Previous studies have reported that NF-*κ*B-p65 phosphorylation at Ser-536 contributes to the inhibition of p53 transcriptional activity and subsequent anti-apoptotic effects.^27, 28^ Our results demonstrated that VD with TNF-*α* treatment significantly decreased expression of NF-*κ*B-p65 and NF-κB-p65 (phospho S536) and induced a p53-mediated pro-apoptotic effect in RA-FLSs.

Whether Sirt1 acted to mediate anti-apoptosis or pro-apoptosis pathogenic responses in RA-FLSs is uncertain. Previous studies demonstrated that Sirt1 overexpression promoted apoptotic resistance and inflammatory secretion in RA-FLSs;^25^ Sirt1 gene silencing promotes apoptosis of RA-FLSs.^29^ In contrast, evidence suggests that Sirt1 is important in anti-inflammation through downregulating expression of the cysteine-rich protein 61 in RA-FLSs;^30^ resveratrol, a Sirt1 activator, induces apoptosis of RA-FLSs.^31^ Recent studies report that subcellular localization of Sirt1 is associated with cell fate. Nuclear-localized Sirt1 is involved in anti-apoptosis, whereas cytoplasmically localized Sirt1 promote apoptosis that is independent of de-acetylation but dependent on Caspase signaling.^32, 33^ Moreover, overexpression and nuclear-localization of Sirt1 significantly promotes apoptotic resistance in RA-FLSs.^25^ Thus, the subcellular localization of Sirt1 in FLSs may be pivotal to RA progression. Our results demonstrated that TNF-*α* treatment inhibited translocation of Sirt1 from nuclei to cytoplasm; VD with TNF-*α* treatments promoted Sirt1 translocation from nuclei to cytoplasm in human rheumatoid FLSs. However, further investigation is needed to determine if cytoplasm-localized Sirt1 is necessarily associated with pro-apoptosis in RA-FLSs and the exact regulating mechanism.

In conclusion, the ‘*in vivo*' and ‘*in vitro*' data presented in this paper strongly suggest that VD with TNF-*α* protected against RA by promoting apoptosis of RA-FLSs. Our findings implied that clinical administration of VD could be as a specific therapy to prevent RA progression.

## Materials and Methods

### Mice and genotyping

Adult *CYP27B1* heterozygote (*CYP27B1*^+/−^) mice (129sv/J hybrid background) were backcrossed 10–12 times to the BALB/c background and mated to generate *CYP27B1* homozygotes (*CYP27B1*^−/−^) and their wild-type (WT) littermates. Mice were genotyped by PCR as described previously.^34, 35^ Male *CYP27B1*^−/−^ and WT mice were used at 10 weeks of age. Mice were weaned at 3 weeks and fed rescue diet (TD96348 Teklad, Madison, WI, USA) containing 2% calcium, 1.25% phosphorus and 20% lactose until they were 19 weeks old. We confirmed that serum calcium and phosphorus levels were normalized in *CYP27B1*^−/−^ mice and WT littermates fed with rescue diet (Supplementary Table S1).

This study was carried out in strict accordance with the guidelines of the Institute for Laboratory Animal Research of Nanjing Medical University in Nanjing, China. The protocol was approved by the Committee on the Ethics of Animal Experiments of Nanjing Medical University (Approval ID 20111201).

### Administration of VD *in vivo*

The 10-week-old mice were divided into three groups of VD-treated *CYP27B1*^−/−^ CIA, vehicle-treated *CYP27B1*^−/−^ CIA or WT CIA mice. For 9 weeks, animals received intraperitoneal treatment of vehicle or 1 *μ*g/kg VD (Sigma-Aldrich, St. Louis, MO, USA) every other day.

The 10-week-old male WT mice derived from BALB/c background were divided into two groups of VD-treated and vehicle-treated mice. For 3 weeks, animals received intraperitoneal treatment of vehicle or 1 *μ*g/kg VD every other day.

### Measurements of serum calcium, phosphorus and VD

Mice were anesthetized with 3% pentobarbital sodium (40 mg/kg) and the abdomen depilated. Blood was taken by suction from the heart with a 1 ml syringe.

Serum calcium and phosphorus levels were analyzed by an autoanalyzer (Beckman Synchron67; Beckman Instruments, Fullerton, CA, USA) from 10-week-old *CYP27B1*^−/−^ mice and WT littermates.

Serum VD was measured by radioimmunoassay (Diagnostic Products, Los Angeles, CA, USA) from 10-week-old *CYP27B1*^−/−^ mice and WT littermates, 19-week-old VD-treated *CYP27B1*^−/−^, vehicle-treated *CYP27B1*^−/−^ and WT CIA mice, and 13-week-old VD-treated and vehicle-treated WT mice.

### Induction and assessment of collagen-induced arthritis

CIA was induced as previously described.^9, 14, 36^ Immunization-grade chicken type II collagen (Chondrex Inc., Redmond, WA, USA) was dissolved in 0.1 mM acetic acid (2 mg/ml) at 4 °C overnight and emulsified with an equal volume of complete Freund's adjuvant ((Sigma-Aldrich) on ice. Male *CYP27B1*^−/−^ and WT littermates at 10 weeks of age were injected subcutaneously at the tail base with 100 *μ*l emulsion, which was considered the primary immunization on Day 1. Secondary immunization on Day 22 was similar to primary immunization on Day 22. Mice were analyzed by three independent, blinded examiners every sixth day from Day 7 and monitored for signs of arthritis onset using clinical parameters of cumulative arthritis scores for subjective evaluation of arthritis severity. Clinical arthritis was scored as previously described:^9^ 0, no evidence of erythema and swelling; 1, erythema and mild swelling confined to the tarsals or ankle joint; 2, erythema and mild swelling extending from the ankle to the tarsals; 3, erythema and moderate swelling extending from the ankle to metatarsal joints; and 4, erythema and severe swelling encompassing the ankle, foot and digits, or ankylosis of the limb. Paw swelling was assessed by measuring the thickness of affected hind paws with 0–10 mm calipers.^36^

### Rheumatoid serum biochemical measurements

At 19 weeks, mice were anesthetized with 3% pentobarbital sodium (40 mg/kg) and the abdomen depilated. Blood was taken by suction from the heart with a 1 ml syringe. Serum was isolated for measurements of RFs (E035 RF detection kit) and C-RP (E023 C-RP detection kit) according to the manufacturers' instructions (Nanjing Jiancheng Bioengineering Institute, Nanjing, China).

### Histological analysis

Phenotypes of 19-week-old VD-treated *CYP27B1*^−/−^ CIA mice were compared with vehicle-treated *CYP27B1*^−/−^ CIA and WT CIA mice.

Mice were anesthetized with 3% pentobarbital sodium (40 mg/kg) and perfused with 100 ml normal sodium and then fixed with periodate–lysine–paraformaldehyde (PLP) solution.^37^ Hind palms were dissected.

For histochemistry or immunohistochemistry, samples were decalcified and dehydrated in a series of graded ethanol solutions, embedded in paraffin and cut into 5-*μ*m sections with rotary microtome (Leica Microsystems Nussloch GmbH, Nubloch, Germany).^38^

### Skeletal radiography and micro-computed tomography

Hind palms were removed and dissected free of soft tissue. Contact radiographs were taken using a Faxitron Model 805 radiographic inspection system (Faxitron Contact, Faxitron, Germany; 22 kV and 4-min exposure) as described previously.^38, 39^ Micro-computed tomography (CT) used a SkyScan 1072 scanner and analysis software (SkyScan, Antwerp, Belgium) as described previously.^38, 39^ Image acquisition was at 100 kV and 98 mA with a 0.9-degree rotation between frames. Two-dimensional images were used to generate three-dimensional renderings using 3D Creator software supplied with the instrument. Micro-CT image resolution is 9 *μ*m.^38, 39^

### Histochemical or immunohistochemical staining

Sections were stained with hematoxylin and eosin (HE), and total collagen labeled by picrosirius red, or Safranin O and fast green double dyeing (SO-FG) (Sigma-Aldrich) as previously described.^38, 40^

Immunohistochemical staining was performed as described previously.^37, 38, 41^ Serial paraffin sections were deparaffinized and dehydrated. For antigen retrieval, sections were steamed for 10–15 min in IHC Antigen Retrieval Solution (10 ×) (eBioscience Inc., San Diego, CA, USA) dissolved in deionized water followed by blocking of endogenous peroxidase (3% H_2_O_2_) and preincubation with serum. Primary antibodies against TNF-*α*, interleukin (IL)-1*β*, CD3, F4/80 (Santa Cruz Biotechnology, Inc., Dallas, TX, USA), PCNA (Dako Cytomation Denmark A/S, Glostrup, Copenhagen, Denmark), Caspase3 and Sirt1 (Cell Signaling Technology, Beverly, MA, USA) were used. After washing, sections were incubated with secondary antibody (biotinylated immunoglobulin G; Sigma-Aldrich), washed, and processed using Vectastain ABC-HRP kits (Vector Laboratories Inc., Burlingame, CA, USA). Sections were counterstained with hematoxylin and mounted with biomount medium.^37, 38^ For immunofluorescence, affinity-purified Alexa Fluor 488-conjugated secondary antibody (Life Technologies Corporation, Carlsbad, CA, USA) was used. Nuclei were labeled by DAPI (Sigma-Aldrich) and mounted with medium to prevent quenching (Vector Laboratories Inc.).^38, 41^

### TUNEL assay

Dewaxed and rehydrated paraffin sections were stained with an *In Situ* Cell Death Detection Kit (Roche Diagnostics Corp., Basel, Switzerland) using a previously described protocol.^37, 38^

### Cell lines and cell cultures

MH7A (human rheumatoid FLSs) and normal FLSs (American Type Culture Collection, Manassas, VA, USA) were cultured in DMEM supplemented with 10% FBS (Gibco, Carlsbad, CA, USA), 100 U/ml penicillin and 100 *μ*g/ml streptomycin (Gibco) at 37°C in a humidified atmosphere of 5% CO_2_ as previously described.^14^

### Administration of VD, TNF-*α* or PFT-*α in vitro*

MH7A cells were treated with DMEM for 24 h, then with DMEM and 10^–6^, 10^–7^, 5 × 10^–8^, 10^–8^ or 10^–9^ M VD (Sigma-Aldrich) for 48 h.

MH7A cells were treated with DMEM for 24 h, then with DMEM and 5, 10, 20, 30 or 40 ng/ml TNF-*α* (Novoprotein Scientific Inc., Shanghai, China) treatment for 48 h. MH7A cells were treated with DMEM for 24 h, then with DMEM and 5, 10, 20, 30 or 40 *μ*M pifithrin-*α* (PFT-*α*) (Sigma-Aldrich) (a p53 pro-apoptotic inhibitor) for 48 h.

Normal FLSs were treated with DMEM for 72 h or DMEM and 10^–7^ M VD and/or 10 or 30 ng/ml TNF-*α* (Sigma-Aldrich) for 24 h.

### Small interference RNA-mediated knockdown of human Vitamin D receptor

For small interference RNA (siRNA) experiments, RNA primers complementary to human VDR were designed and synthesized (Ribobio Co. Ltd., Guangzhou, China). siRNAs were transfected using Lipofectamine 2000 reagent (Invitrogen, Carlsbad, CA, USA) according to the manufacturer's instructions.^37^ After incubation for 72 h, MH7A cells were harvested and mRNA was detected by real-time RT-PCR. Sequences of siRNAs and targeted mRNAs are in Supplementary Table S2.

### Cytotoxicity tests by cell counting Kit-8 assays

MH7A cells (5 × 10^4^ per ml, 100 *μ*l) were plated into 96-well plates with five parallel wells per group. After 12 h in DMEM with FBS, cells were treated with DMEM without FBS for 24 h, then with indicated concentrations of VD, TNF-*α*, PFT-*α*, human VDR siRNA, or negative control (NC) and positive control (PC) siRNA. After 48 h, cell viability was determined by cell counting kit-8 (CCK-8) assay kits (Lot C0038) (Beyotime Institute of Biotechnology, Shanghai, China) and detected by spectrophotometry at 450 nm absorbance following manufacturer's instructions.^42^

Viability of cells treated with VD, TNF-*α*, PFT-*α*, human VDR siRNA, NC and PC siRNA was normalized to the serum-free control (Supplementary Figure S1B).

### Flow cytometry for apoptosis

Percentage of apoptotic cells was determined by flow cytometry using FITC Annexin V Apoptosis Detection Kits I (Lot 556547) (BD Biosciences, San Jose, CA, USA) following manufacturer's instructions. The number of cells stained with annexin V (AV)–fluorescein isothiocyanate (FITC) and propidium iodide (PI) was assessed by flow cytometer (BD FACScan, Franklin Lakes, NJ, USA). Early apoptotic cells were defined as positive for AV–FITC but negative for PI staining; late apoptotic cells were positive for both AV–FITC and PI; and total apoptotic cells were positive for AV and positive or negative for PI.^43^

### Immunocytochemical staining

For immunocytochemical staining, cells seeded on coverslips were fixed with PLP solution for 45 min and pre-incubated with serum. Primary antibodies against Sirt1 (Cell Signaling Technology) and affinity-purified Alexa Fluor 488-conjugated secondary antibody (Life Technologies Corporation) were used. Nuclei were labeled with DAPI (Sigma-Aldrich) and mounted with medium to prevent quenching (Vector Laboratories Inc.).^37, 38^

### RNA isolation and Real-time RT-PCR

RNA was isolated from MH7A cells using TRIzol reagent (Invitrogen) according to the manufacturer's protocol. Sample mRNA levels were quantified by real-time RT-PCR and calculated as ratio to GAPDH mRNA as previously described.^37, 41^ PCR primers are in Supplementary Table S3.

### Western blots

Western blots using MH7A cells were performed as previously described.^37^ Nuclear and cytoplasm fractions of MH7A cells were extracted using NE-PER Nuclear and Cytoplasmic Extraction Reagents (Pierce Biotechnology, Rockford, IL, USA) according to the manufacturer's protocol. Primary antibodies were against Caspase3 (Cell Signaling Technology), Bax (Santa Cruz Biotechnology), Puma (Abcam, Cambridge, MA, USA), Bcl-2 (Santa Cruz Biotechnology), p53 (Cell Signaling Technology), p53(acetyl K382) (Abcam), NF-*κ*B p65 (Cell Signaling Technology), NF-*κ*B-p65 (phospho S536) (Abcam), Sirt1 (Cell Signaling Technology), VDR (Santa Cruz Biotechnology), Laminin *β*1 (Abcam) or *β*-actin (Bioworld Technology, St. Louis Park, MN, USA). Laminin *β*1 was the loading control for the nuclear fraction and *β*-actin for the cytoplasm fraction and total cell protein.

### Statistical analysis

All analyses were performed using SPSS software (Version 16.0, SPSS Inc., Chicago, IL, USA) as previously described.^37, 38^ Measurement data were described as mean±S.E.M. fold-change over control and analyzed by Student's *t*-test and one-way ANOVA to compare differences among groups. Qualitative data were described as percentages and analyzed using chi-square tests as indicated. *P*-values were two-sided and less than 0.05 was considered statistically significant.

## Figures and Tables

**Table 1:**
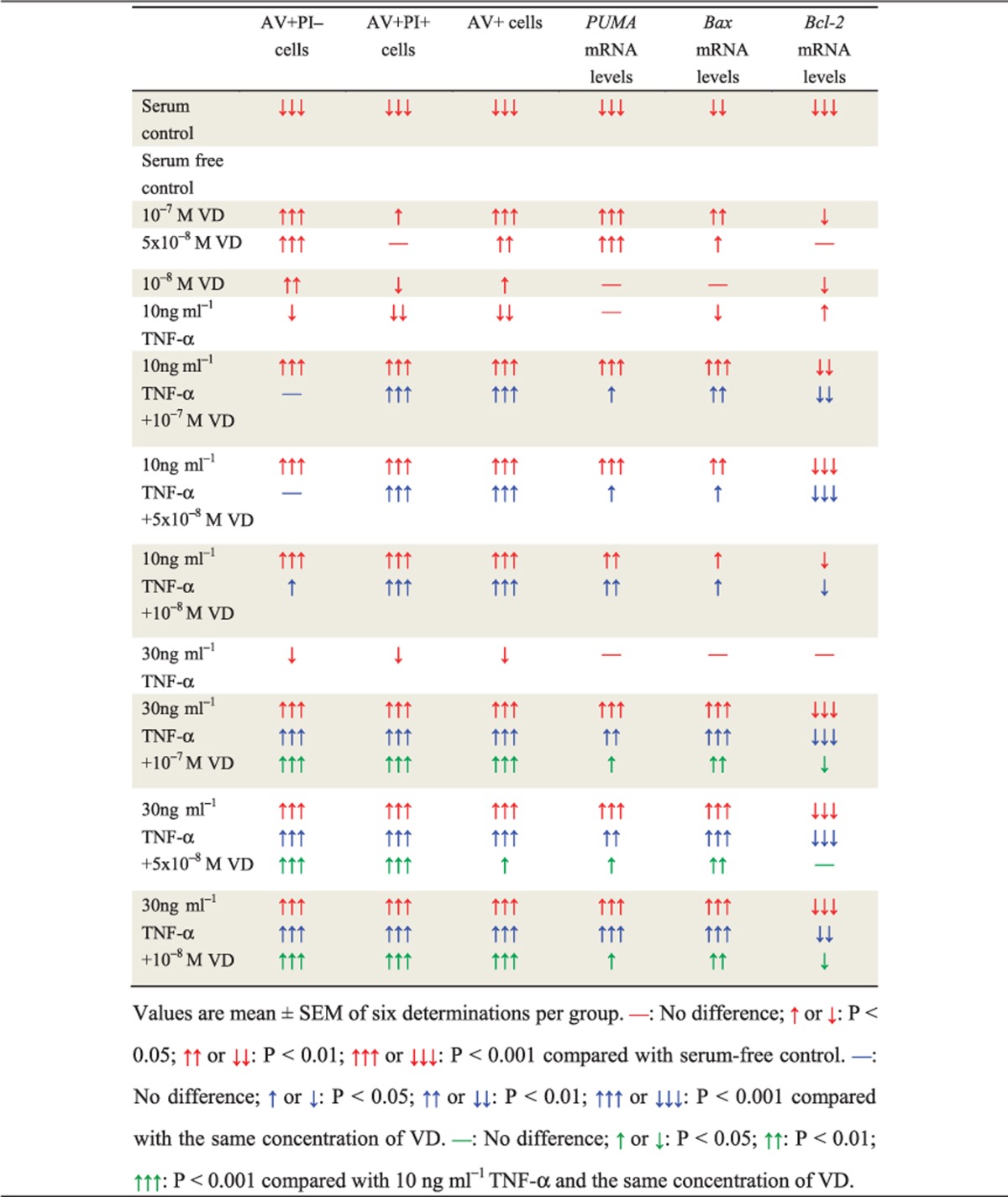
VD with TNF-*α* promoted apoptosis of rheumatoid FLSs

**Table 2:**
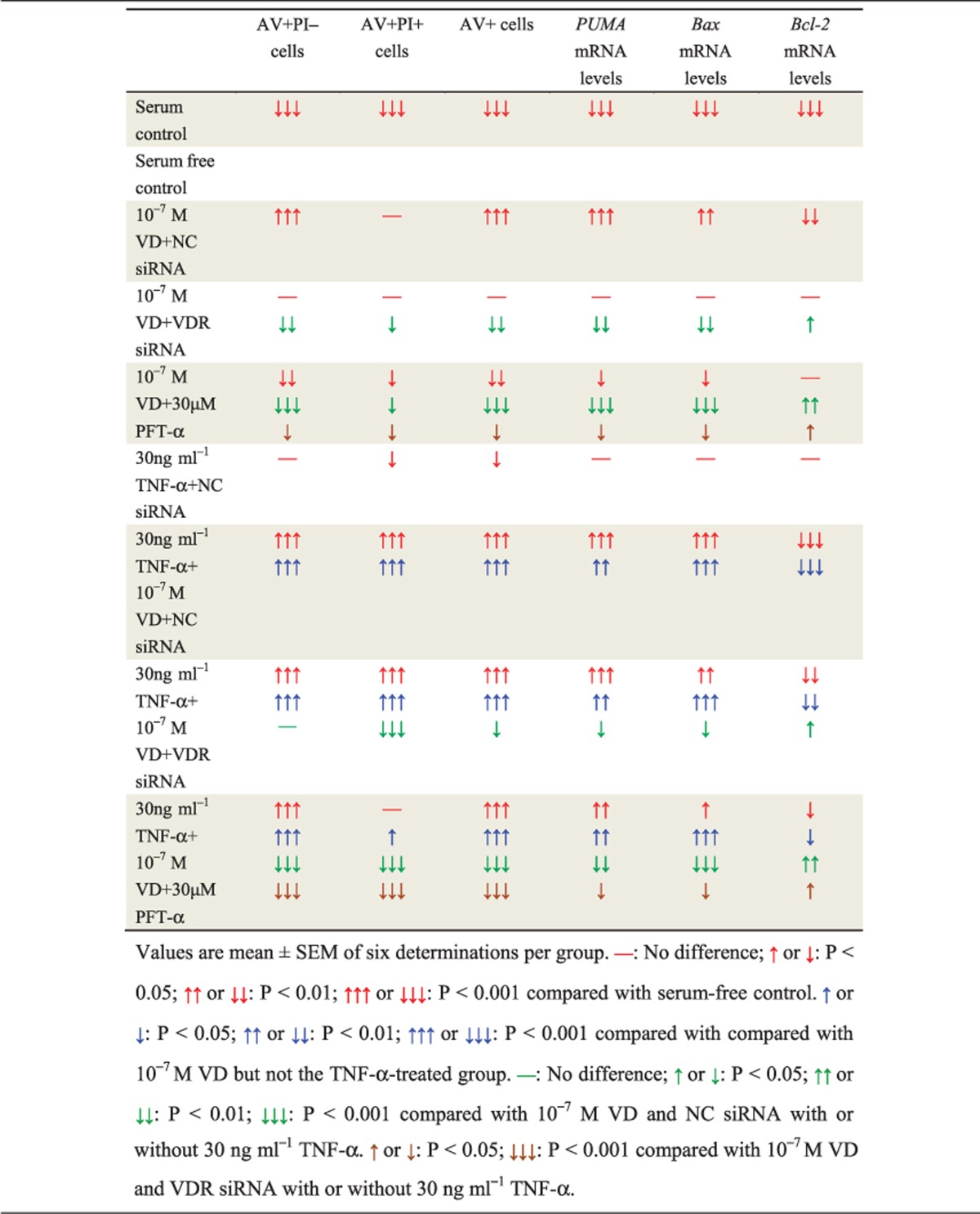
VD with TNF-*α* induced apoptosis of rheumatoid FLSs through VDR and p53 pro-apoptotic signaling

**Table 3:**
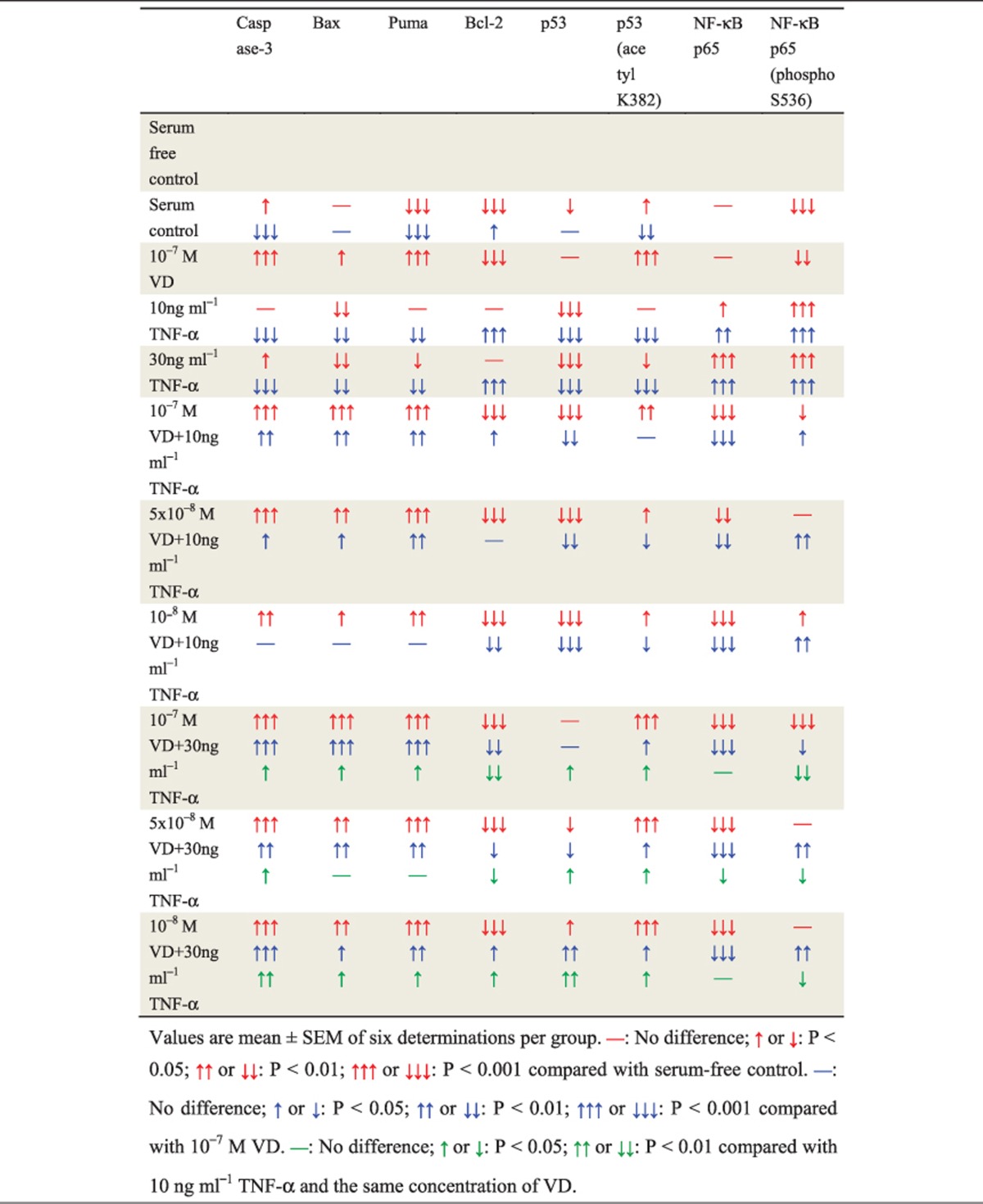
VD with TNF-*α* promoted p53 acetylation-mediated apoptosis in human rheumatoid FLSs

## Abbreviations

VD: 1, 25-dihydroxy-vitamin D3
RA: rheumatoid arthritis
TNF-α: tumor necrosis factor-α
FLSs: fibroblast like synoviocytes
