# Supplementary figures and images for: 1, 25-dihydroxy-vitamin D3 with tumor necrosis factor-alpha protects against rheumatoid arthritis by promoting p53 acetylation-mediated apoptosis via Sirt1 in synoviocytes
Source: Cell Death Dis. 2016 Oct 20;7(10):e2423–. doi: 10.1038/cddis.2016.300 (PMC5133971; doi:10.1038/cddis.2016.300)

Figure S1

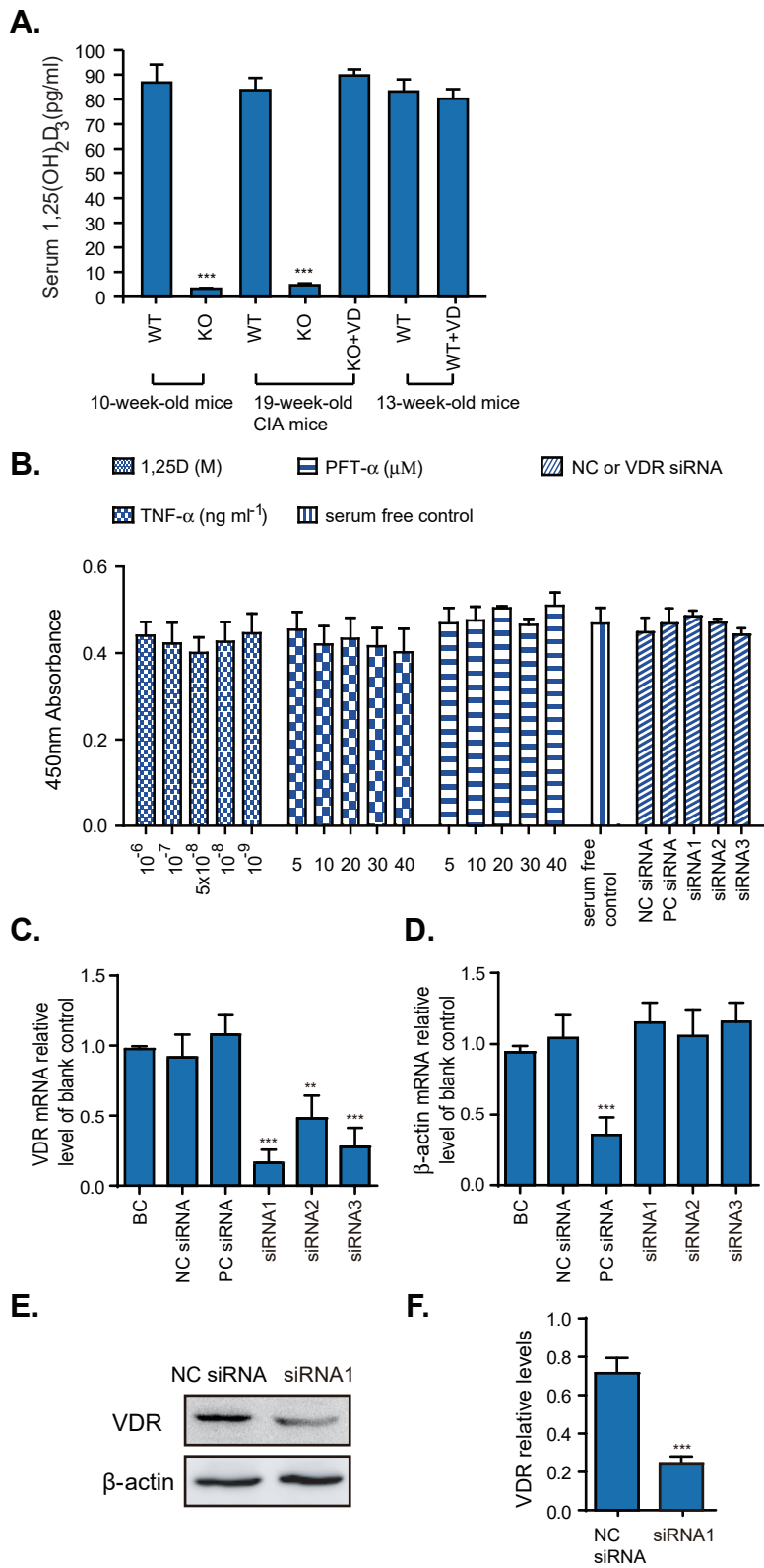

Supplement: Supplementary Figure S1 [file cddis2016300x1.pdf]

Figure S2

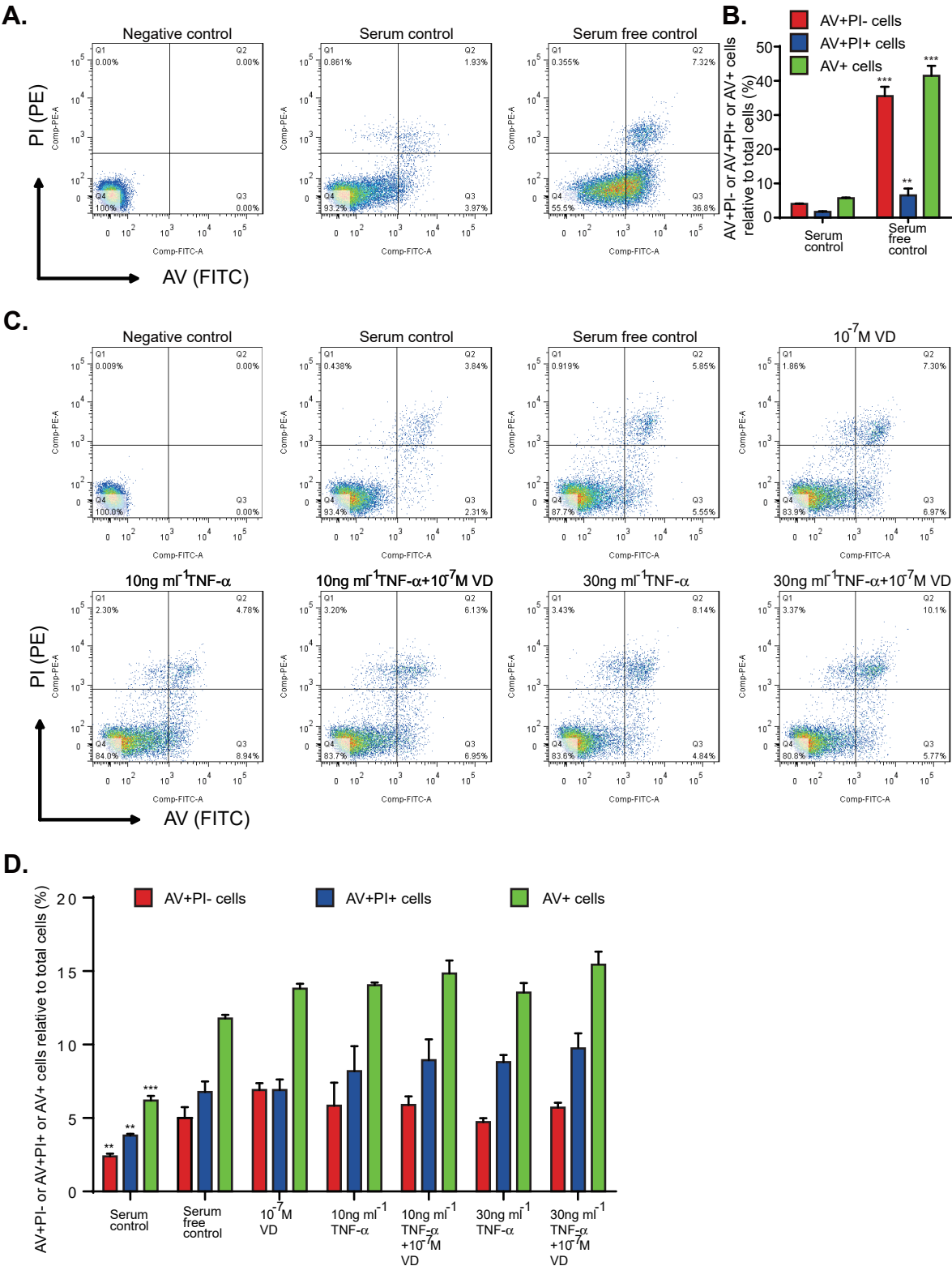

Supplement: Supplementary Figure S2 [file cddis2016300x2.pdf]

Figure 8

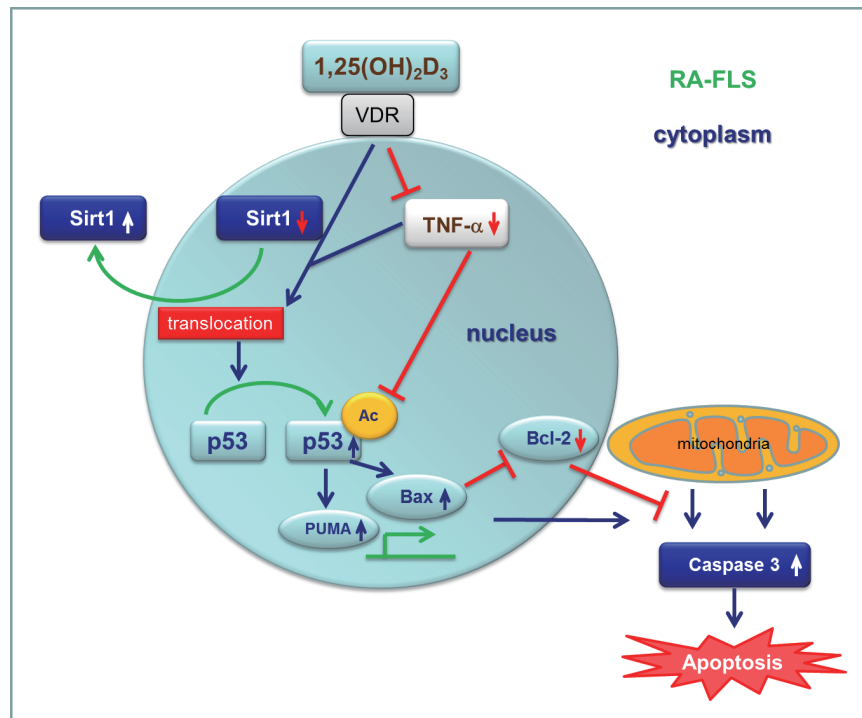

Supplement: Supplementary Figure S3 [file cddis2016300x3.pdf]
