# Supplementary material for: 1, 25-dihydroxy-vitamin D3 with tumor necrosis factor-alpha protects against rheumatoid arthritis by promoting p53 acetylation-mediated apoptosis via Sirt1 in synoviocytes
Source: Cell Death Dis. 2016 Oct 20;7(10):e2423–. doi: 10.1038/cddis.2016.300 (PMC5133971; doi:10.1038/cddis.2016.300)
Supplement: Supplementary legends S1-S2 [file cddis2016300x4.docx]

**Supplementary figure legends**

**Figure S1 Serum VD detection, and cytotoxicity tests** **and human VDR mRNA in MH7A cells knocked down by VDR siRNA in human rheumatoid FLSs.** (**A**) Serum VD was measured by radioimmunoassay from 10-week-old *CYP27B1^-/-^* mice and WT littermates, 19-week-old VD-treated *CYP27B1^-/-^*, vehicle-treated *CYP27B1^-/-^* and WT CIA mice, and 13-week-old VD-treated and vehicle-treated WT mice. Values are mean ± SEM of six determinations per group. ***: P < 0.001 compared with 10-week-old WT or 19-week-old WT CIA mice. (**B**) Cytotoxicity tests with indicated concentrations of VD, TNF-α and pifithrin (PFT)-α, human VDR siRNA, negative control (NC) siRNA and positive control (PC) siRNA were determined by CCK-8 assay and spectrophotometry at 450 nm. (**C**) Human VDR mRNA in MH7A cells from blank control (BC), NC siRNA and siRNA 1−3-transfected cells determined by real-time RT-PCR. (**D**) Human β-actin mRNA in MH7A cells from BC, NC siRNA, PC siRNA and siRNA1−3-transfected cells determined by real-time RT-PCR with mRNA calculated as ratio to GAPDH mRNA, expressed relative to BC. Values are mean ± SEM of six determinations per group. **: P < 0.01, ***: P < 0.001 compared with BC. (**E**) Western blots of cell lysates from NC siRNA and siRNA1-transfected cells for VDR. β-actin was the loading control. (**F**) Protein relative to β-actin was assessed by densitometry. Values are mean ± SEM of six determinations per group. ***: P < 0.001 compared with NC siRNA.

**Figure S2 VD with TNF-α did not promote apoptosis of normal FLSs.** Human normal FLSs were treated with DMEM and 10% FBS (serum control), DMEM (serum-free control), DMEM and indicated concentrations of VD with or without TNF-α. (**A**) Normal FLSs were treated with serum-free culture for 72 h as the MH7A treatment in Figure 4A. Flow cytometry of double-stained cells using annexin V (AV) and propidium iodide (PI). (**B**) AV-positive but PI-negative cells (AV+PI−), AV and PI double-positive cells (AV+PI+), and total AV-positive cells (AV+) were quantified. Values are mean ± SEM of six determinations per group. ***: P < 0.001 compared to serum control. (**C**) Normal FLSs were treated with DMEM or with DMEM and 10^–7^ M VD and/or 10 or 30 ng ml^–1^ TNF-α for 24 h. Flow cytometry of double-stained cells using AV and PI. (**D**) AV+PI−, AV+PI+, and AV+ NFLS cells were quantified. Values are mean ± SEM of six determinations per group. ***: P < 0.001 compared to serum-free control.
