# Supplementary material for: 1, 25-dihydroxy-vitamin D3 with tumor necrosis factor-alpha protects against rheumatoid arthritis by promoting p53 acetylation-mediated apoptosis via Sirt1 in synoviocytes
Source: Cell Death Dis. 2016 Oct 20;7(10):e2423–. doi: 10.1038/cddis.2016.300 (PMC5133971; doi:10.1038/cddis.2016.300)
Supplement: Supplementary Tables S1-S3 [file cddis2016300x5.docx]

**Table S1** The measurement of Serum Calcium or Phosphorus

| Name | WT | *CYP27B1^-/-^* |
| --- | --- | --- |
| Serum Ca^2+^, mM  Serum PO_4_^3-^, mM | 2.54±0.18  3.05±0.25 | 2.47±0.13  2.98±0.21 |

Values are mean ± SEM of six determinations of each group. All tests were done on 10-week-old mice.

**Table S2** siRNAs against Human VDR

| Name | S/AS | Sequence | Target mRNA Sequences |
| --- | --- | --- | --- |
| siRNA 1  siRNA 2  siRNA 3  Negative control siRNA  Positive control siRNA (against human β-actin) | S  AS  S  AS  S  AS  S  AS  S  AS | 5′-CUAAGAUGAUACCAGGAUU dTdT-3′  5′-dTdT GAUUCUACUAUGGUCCUAA-3′  5′-AGCGCAUCAUUGCCAUACU dTdT-3′  5′-dTdT UCGCGUAGUAACGGUAUGA-3′  5′-GUCAGUUACAGCAUCCAAA dTdT-3′  5′-dTdT CAGUCAAUGUCGUAGGUUU-3′  —  —  —  — | CTAAGATGATACCAGGATT  AGCGCATCATTGCCATACT  GTCAGTTACAGCATCCAAA  —  — |

S, sense; AS, antisense

**Table S3** Primers for Real Time RT-PCR

| Name | S/AS | Sequence | Species | Tm  (°C) | Length  (bp) |
| --- | --- | --- | --- | --- | --- |
| *VDR*  *GAPDH*  *β-actin*  *PUMA*  *Bax*  *Bcl-2* | S  AS  S  AS  S  AS  S  AS  S  AS  S  AS | 5′-TCTCCAATCTGGATCTGAGTGAA-3′  5′-GGATGCTGTAACTGACCAGGT-3′  5′-GTGTGAACCATGAGAAGTATGAC-3′  5′-CTGGGTGGCAGTGATGGCATGGAC-3′  5′-TCAAGATCATTGCTCCTCCTGAG-3′  5′-ACATCTGCTGGAAGGTGGACA-3′  5′-ATGGCGGACGACCTCAAC-3′  5′-AGTCCCATGAAGAGATTGTACATGAC-3′  5′-CCCGAGAGGTCTTTTTCCGAG-3′  5′-CCAGCCCATGATGGTTCTGAT-3′  5′-GGTGGGGTCATGTGTGTGG-3′  5′-CGGTTCAGGTACTCAGTCATCC-3′ | human  human  human  human  human  human | 60  60  60  60  61  61 | 111  337  87  168  155  89 |

S, sense; AS, antisense, sequence; Tm, annealing temperature; length, amplicon
